# Plasma exosome-derived connexin43 as a promising biomarker for melanoma patients

**DOI:** 10.1186/s12885-023-10705-9

**Published:** 2023-03-14

**Authors:** Yue Shen, Ming Li, Li Liao, Suyue Gao, Yongzhen Wang

**Affiliations:** 1 Department of Dermatology, Suzhou Municipal Hospital, Gusu School, The Affiliated Suzhou Hospital of Nanjing Medical University, Nanjing Medical University, 242 Guangji Road, Suzhou, Jiangsu, 215008 China; 2 Department of Pathology, Suzhou Municipal Hospital, Gusu School, The Affiliated Suzhou Hospital of Nanjing Medical University, Nanjing Medical University, 242 Guangji Road, Suzhou, Jiangsu, 215008 China; 3 Department of Clinical Laboratory, The Affiliated Suzhou Hospital of Nanjing Medical University, 26 Daoqian Street, Suzhou, Jiangsu, 215008 China

**Keywords:** Connexin 43, Exosomes, Melanoma, Prognosis, Biomarker

## Abstract

**Background:**

To examine the levels of exosome-derived connexin 43 (Cx43) in plasma and estimate its forecast value in patients with melanoma.

**Methods:**

We measured the plasma exosome-derived Cx43 levels in the plasma of 112 melanoma patients and 50 healthy controls.

**Results:**

The plasma exosome-derived Cx43 levels in patients with melanoma were substantially downregulated as opposed to the levels in healthy controls (*P* < 0.001). Kaplan–Meier analysis indicated that overall survival (OS) and disease-free survival (DFS) were poorer in patients with melanoma who exhibited lower levels of plasma exosome-derived Cx43 (both *P* < 0.001). The levels of plasma exosome-derived Cx43 were considerably elevated in patients with melanoma whose tumor was situated in the skin, tumor size < 10 cm, with Clark level I–III, TNM stages IIb–IV, and had no lymph node metastasis as opposed to patients whose tumor was situated in the viscera or mucosa, tumor size ≥ 10 cm, Clark level IV–V, TNM stages IIb–IV and had lymph node metastasis (all *P* < 0.05). The receiver operating characteristic (ROC) of plasma exosome-derived Cx43 for forecasting 5-year DFS in patients with melanoma was 0.78 (95% confidence interval (CI): 0.70–0.86), with a specificity of 77.78% and a sensitivity of 81.55%. The ROC of plasma exosome-derived Cx43 for forecasting 5-year OS of patients with melanoma was 0.77 (95% CI: 0.68–0.84), with a specificity of 80.0% and sensitivity of 65.98%.

**Conclusion:**

The overall findings indicated that the levels of plasma exosome-derived Cx43 in patients with melanoma were considerably downregulated. It can therefore be inferred that the levels of plasma exosome-derived Cx43 might be a prospective prognostic indicator for 5 5-year OS and 5-year DFS of patients with melanoma.

**Supplementary Information:**

The online version contains supplementary material available at 10.1186/s12885-023-10705-9.

## Introduction

Melanoma has been extensively recognized as a highly malignant tumor originating from the melanocytes in the basal layer of the epidermis [1, 2]. Most melanomas occur in the skin, but also in the mucosa and viscera, and account for about 3% of all tumors. Malignant melanoma has been found to be the third commonest type of skin malignancy (6.8–20%), and is more common in adults, especially in fair-skinned Caucasians [3, 4]. Over the past few years, the prevalence and fatality of malignant melanoma have been increasing progressively, with an annual growth rate of 3–5% [5]. It has been reported that skin malignant melanoma has gene mutations, such as *BRAF, CKIT, and NRAS* [6]. Clinical studies have shown that molecular targeted therapy is the main strategy for metastatic melanoma, although only a limited proportion of patients can enjoy its benefits [7]. Consequently, there is no specific treatment for most patients with metastatic melanoma. Early surgical resection is the preferred treatment for malignant melanoma, but the prognosis of patients is poor and the postoperative survival is generally not optimistic [8].

Connexin (Cx) is a member of the transmembrane protein family. More than 20 kinds of connexins have been found in humans, among which, Cx43 is the most common [9, 10]. Cx43 is an abundant and widely distributed junction protein, which is mainly distributed on the surface of cell membranes in normal tissues. It is mainly involved in cell gap junction communication, providing a necessary channel for information exchange between cells, and also providing a connection pathway for tumor cells and vascular endothelial cells [11, 12]. In addition, the function of gap junction channels is regulated by the expression of CX43, and both quantitative and qualitative changes of gap junction proteins can influence the occurrence, differentiation, invasion as well as metastasis of tumors [13]. In recent years, CX43 has been found to be strongly linked to the occurrence as well as the development of many malignant tumors. Bišćanin et al. found that Cx43 was more highly expressed in normal tissues compared with colorectal cancer tissues, suggesting that Cx43 has antitumor properties [14]. Teleki et al. found that gap junction proteins Cx26, Cx30, Cx32, Cx43, and Cx46 were differentially expressed in breast cancer progression and prognosis, and significant correlations were also found at the mRNA level. Elevated levels of Cx43 significantly improve the prognosis of breast cancer [15]. Compared to reliance on vascular invasion or necrosis as an evaluation index, Cx43 protein detection is more valuable as an independent prognostic indicator. High expression of Cx43 is linked to reduced overall survival (OS) in patients with oral squamous cell carcinoma [16]. Cx43’s function in the production of vascular endothelial growth factor (VEGF) and tumor angiogenesis in mice suggests that reduced Cx43 expression leads to increased expression of VEGF in B16F10 melanoma cells and 4T1breast cancer cells, which promotes tumor metastasis [17]. However, overexpression of Cx43 can reduce angiogenesis and inhibit tumor growth.

Exosomes are membrane-covered vesicles secreted by a variety of living cells, which contain proteins, lipids, nucleic acids, and a variety of other biological macromolecules [18–20]. Exosomes perform a vital function in many pathophysiological processes, including antigen presentation in the immune response, repair of tissue damage, and tumor growth and migration [21, 22]. Tumor-derived or tumor-related exosomes have significant implications for the regulation of tumorigenesis and development. Analysis and detection of tumor exosomes can assist in the early diagnosis of tumors and provide new treatment methods for patients [23–27].

A growing body of research shows that exosomes comprise of numerous essential proteins that might be utilized for early tumor diagnosis, patient prognosis analysis, and tumor-targeting treatment [28–30]. In this research, we intended to examine the plasma exosome-derived Cx43 level and determine the prognostic value of patients with melanoma.

## Materials and methods

### Patients

We collected blood specimens from 112 patients with melanoma patients from the Suzhou Municipal Hospital, from October 2011 to March 2016. All patients were diagnosed with malignant melanoma by surgical resection and pathology; the course of the disease is 1 ~ 2 years, and there is no treatment. Exclusion criteria: patients treated with surgery, radiotherapy, and chemotherapy; Patients with other malignant tumor diseases; Insanity and contraindications to the study drugs [31]. The patients’ follow-up occurred for a median duration of 65.5 months (range: 13.0–119.0 months) ending in March 2021. The statistics for survival were collected from follow-up records, and DFS and OS were computed.

During the same period, specimens from 50 healthy subjects (with a median age of 64 years, ranging from 48 to 72 years) were taken from the Suzhou Municipal Hospital as healthy controls. After receiving the informed consent of patients or their families and the subjects provided formal informed consent, all specimens were included in the experiment. Approval of the research protocol was gotten from the Ethics Committees of the Suzhou Municipal Hospital, (identification nos. HMU [Ethics] 2022 − 120).

### Plasma exosome isolation

The plasma samples were taken out at -80℃, centrifuged at 4℃ for 2000 g/min for 10 min in order to remove cell fragments, and then centrifugated at 4℃ for 10,000 g/min for 30 min so as to remove macromolecular impurities. The exosome was purified with Beckmann over speed centrifuge at 4℃ at 110,000×g for 70 min, and then the final concentration of 8 mol/L urea was added. A protease inhibitor was added (lysate: protease inhibitor: 50:1). Samples were mixed, ultrasonic cracked, centrifuged at 14,000×g at 4℃ for 20 min. We then performed protein quantification and SDS-PAGE quality control.

### Transmission electron microscopy (TEM)

The exosomes were diluted and filtered. The sample of 5uL was taken and dropped on the copper mesh followed by incubation at an ambient temperature for 5 min. After incubation, we used absorbent paper to drain excess liquid on one side, and then added a drop of 2% uranyl acetate to the copper mesh ensued by incubation at an ambient temperature for 1 min. After incubation, we drained excess liquid on one side with absorbent paper and dried it at room temperature for about 20 min. Finally, we observed the appearance of exosomes utilizing an electron microscope, captured images, and recorded.

### Nanoparticle tracking analysis (NTA)

The specimens were frozen and then thawed in a water bath at a temperature of 25 °C before being placed on ice. Exosome specimens were diluted in 1x PBS before being utilized to detect NTA (ZetaVIEW S/N 17–310). To examine particle mobility and determine the number of exosomes, NTA Software (ZetaView 8.04.02) was utilized.

### Western blotting (WB)

Use a 1.5 mm glass plate and a 15-well sample comb to prepare a 12% separated gel and 5% concentrated gel. Perform the electrophoresis at 80 V of the stabilized voltage until Loading Buffer enters the separating gel, and then continue the electrophoresis at 120 V until the Loading Buffer reaches the bottom of the gel. Select the PVDF membrane with a constant current of 200 mA and membrane transfer time of 90 min. Block the PVDF membrane with PBST diluted 5% skim milk powder for 1 h, wash three times using PBST for 10 min for each washing. Put the membrane into the hybridization box, add the primary antibody, and put it in the decolorizing shaker at 4℃ overnight, including CD63, CD9, and TSG101(Santa Cruz Biotechnology, TX, USA). Remove the primary antibody and wash the membrane with PBST 3 times for 10 min for each wash. Next, add the secondary antibody, place the hybridization box on the shaker, shake slowly and incubate at room temperature for 1 h. Afterward, withdraw the secondary antibody and wash the membrane 3 times with PBST for 10 min during each wash. Add the appropriate amount of ECL luminescent solution and use a digital imaging system to take continuous photos of the membrane.

### ELISA

Diluted the plasma samples with 1×PBS buffer (1:500 dilution). The exosomes should be precipitated with 100 mL RIPA lysate on ice for a duration of 30 min. After shaking and mixing, dilution of the specimens was done using the PBS buffer at a ratio of 1:3. In the Cx43 antibody-coated microtiter plate, the blank and standard control were added. Incubated the 100 µl of the diluted exosomal sample at a temperature of 37 °C for a duration of 1 h, removed the fluid from the microplate by shaking, pat dry, added the solution, incubated again at a temperature of 37 °C for 1 h, then washed 3 times, added solution B, incubated yet again at a temperature of 37 °C for a duration of 30 min, washed 5 times, added 90 µl substrate, incubated at a temperature of 37℃ for a period of 15 min, added 50 µl of termination solution, and immediately measure the absorbance at 450 nm wavelength.

### Statistical analysis

The SPSS 24.0 Software (IBM) was utilized to conduct statistical analyses. Continuous information was displayed as counts (%ages), medians (ranges), or means ± standard deviations. Wilcoxon’s rank-sum tests or Chi-squared tests were conducted for correlation analysis. The log-rank was employed to determine the variations in OS and DFS between two cohorts. The prognostic significance of plasma exosome-derived Cx43 levels was evaluated using a ROC curve analysis. Statistically significant differences were defined as those with a *P* value of less than 0.05.

## Results

### Baseline characteristics of enrolled melanoma patients

Table 1 displays the baseline features of 112 patients with melanoma. There were 59 men (52.68%) and 53 women (47.32%); 64 (57.14%) were ≥ 60 years old while 48 (42.86%) were < 60 years old. In 98 instances (87.50%), the tumor size was < 10 cm, and in 14 cases, it was ≥ 10 cm (12.50%). With regards to tumor location, eighty-six melanomas (76.79%) were found in the skin, whereas twenty-six (23.21%) were found in the viscera or mucosa. Fifty-two melanomas (46.43%) were Clark level I–III and 60 (53.57%) were Clark level IV–V, and 50 (44.64%) were stage 0–IIa, and 62 (55.36%) were stage IIb–IV. There were 63 cases (56.25%) with lymph node metastasis.

**Table 1:** Baseline characteristics of enrolled melanoma patients

Characteristic	Melanoma patients (n = 112)
**Gender**	
Male	59(52.68)
Female	53(47.32)
**Age(years)**	
< 60	48(42.86)
≥ 60	64(57.14)
**Tumor diameter (cm)**	
< 10	98(87.50)
≥ 10	14(12.50)
**Tumor location**	
Skin	86(76.79)
Mucous membrane, viscera	26(23.21)
**Depth of tumor invasion(Clark level)**	
I-III	52(46.43)
IV-V	60(53.57)
**Tumor stage**	
0-IIa	50(44.64)
IIb-IV	62(55.36)
**Lymph node metastasis**	
YES	63(56.25)
NO	49(43.75)

### Characterization of plasma exosomes in melanoma patients

**Fig. 1:**
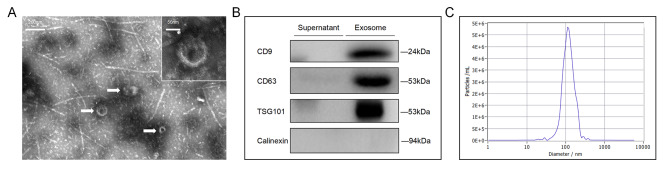
Exosome characterization

The observation results under transmission electron microscopy showed that the background was clear, exosomes aggregated and distributed in the field of vision, with relatively uniform and relatively full size. The diameter ranged from 100 to 200 nanometers. The shape of exosome was a double disc -like vesicle structure with a full lipid envelop. The particle size of exosomes was detected by NTA, and the overall particle median size was around 100 nm, with the majority of the particles distributed between 50 and 200 nm (Fig. 1A). A limited number of particles with diameters ranging from 0 to 50 nm were found, and none had a diameter larger than 300 nm (Fig. 1B). The expression levels of exosomes marker proteins CD63, CD9, and TSG101 were detected by Western blot. The result suggested that CD63, CD9, and TSG101 were positive in the Exo cohort (Fig. 1C). The results indicated that exosomes can be successfully isolated and used in the subsequent experiments of this study.

### The levels of plasma exosome-derived Cx43 in melanoma patients

**Fig. 2:**
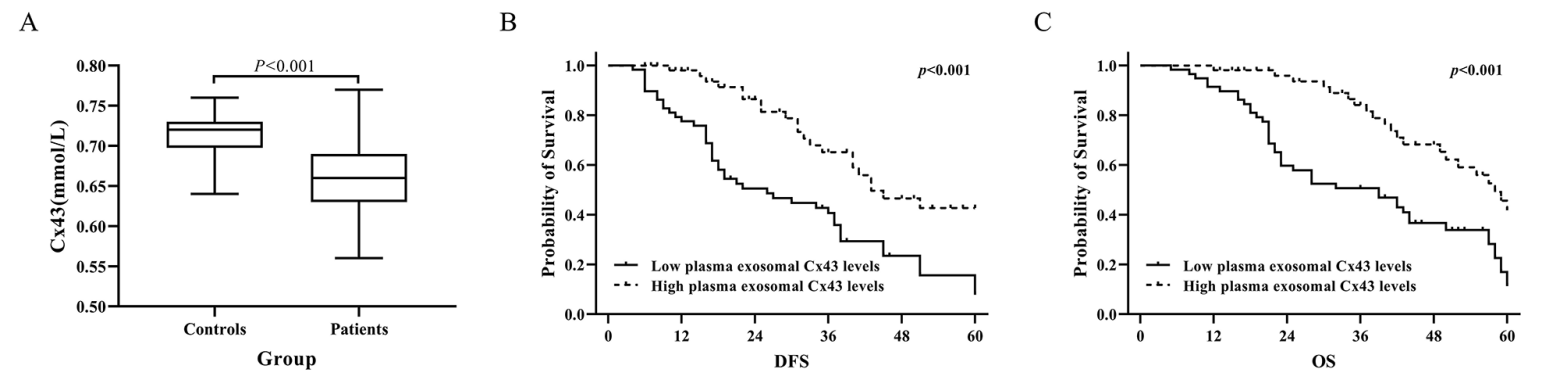
The levels of plasma exosome-derived Cx43 in melanoma patients

As opposed to the plasma exosome-derived Cx43 levels in healthy controls, the levels of plasma exosome-derived Cx43 in patients with melanoma were substantially downregulated (0.66 ± 0.05 mmol/L vs.0.71 ± 0.03 mmol/L, *P* < 0.001; Fig. 2A). Kaplan–Meier (KM) analysis illustrated that OS and DFS were poorer in patients with melanoma who exhibited reduced levels of plasma exosome-derived Cx43 as opposed to the patients who had elevated levels (both *P* < 0.001; Fig. 2B, C).

### Relationship between plasma exosome-derived Cx43 and pathological features in patients with melanoma

**Fig. 3:**
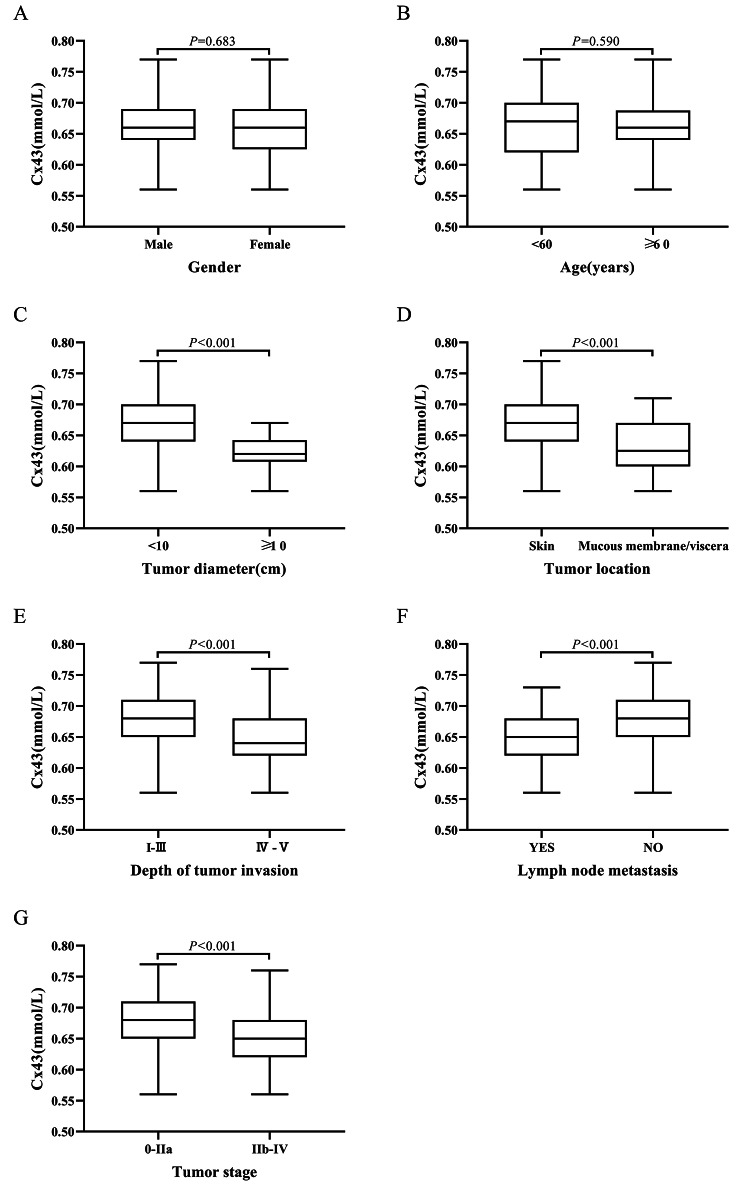
Relationship between plasma exosome-derived Cx43 levels and pathological features in patients with melanoma

There was no significant difference in sex or age in the levels of plasma exosome-derived Cx43 between patients with melanoma and healthy control subjects (both *P* > 0.05; Fig. 3A, B). Nevertheless, the levels of plasma exosome-derived Cx43 in patients with melanoma whose tumor was situated in the skin, with a size of < 10 cm, Clark level I–III, TNM stage IIb–IV, and had no lymph node metastasis were considerably elevated as opposed to patients with a tumor located in the viscera or mucosa, with a size of ≥ 10 cm, Clark level IV–V, TNM stage IIb–IV and had lymph node metastasis (all *P* < 0.05, Fig. 3C–G).

### Prognostic value of plasma exosome-derived Cx43 levels for predicting 5-year DFS and OS of melanoma patients

**Fig. 4:**
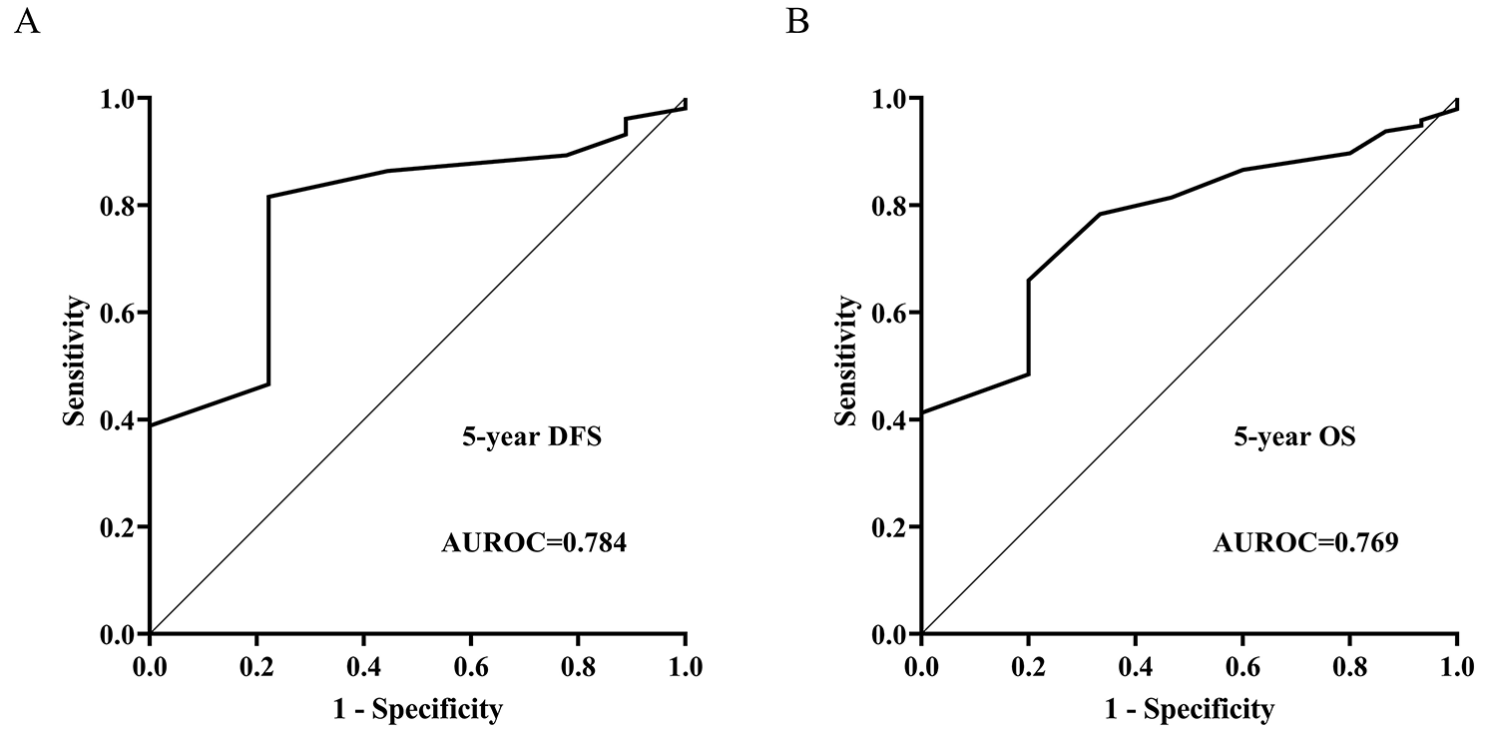
Prognostic value of plasma exosome-derived Cx43 levels for predicting 5-year DFS and OS of melanoma patients

The ROC of plasma exosome-derived Cx43 for forecasting the 5-year DFS of patients with melanoma was 0.78 (95% CI: 0.70–0.86). (Fig. 4A; Table 2). The positive predictive value and positive likelihood ratio were 97.70 (95% CI: 91.90–99.70) and 3.67 (95% CI: 1.10–12.50), sequentially, with a threshold value of 0.69 mmol/L with a specificity of 77.78% (95% CI: 40.0–97.20%) and sensitivity of 81.55% (95% CI: 72.70–88.50%), the negative predictive value and negative likelihood ratio were 26.90 (95% CI: 11.60–47.80) and 0.24 (95% CI: 0.10–0.40), respectively.

**Table 2:** The prognostic value of plasma exosome-derived Cx43 levels in melanoma patients

Variable	prognostic value (n = 112)
**5-year DFS**	
AUROC	0.78(95% CI: 0.70–0.86)
Cutoff value (95%CI)	0.69
Sensitivity, %	81.55(95% CI: 72.70–88.50)
Specificity, %	77.78(95% CI: 40.00-97.20)
Positive predictive value, %	97.70(95% CI: 91.90–99.70)
Negative predictive value, %	26.90(95% CI: 11.60–47.80)
Positive likelihood ratio	3.67(95% CI: 1.10–12.50)
Negative likelihood ratio	0.24(95% CI: 0.10–0.40)
**5-year OS**	
AUROC	0.77(95% CI: 0.68–0.84)
Cutoff value (95%CI)	0.67
Sensitivity, %	65.98(95% CI: 55.70–75.30)
Specificity, %	80.00(95% CI: 51.90–95.70)
Positive predictive value, %	95.50(95% CI: 87.50–99.10)
Negative predictive value, %	26.70(95% CI: 14.60–41.90)
Positive likelihood ratio	3.30(95% CI: 1.20–9.20)
Negative likelihood ratio	0.43(95% CI: 0.30–0.60)

The ROC of plasma exosome-derived Cx43 for forecasting melanoma patients’ 5-year OS was found to be 0.77 (95% CI: 0.68–0.84) (Fig. 4B; Table 2). The positive predictive value and positive likelihood ratio were 95.50 (95% CI: 87.50–99.10) and 3.30 (95% CI: 1.20–9.20), correspondingly, with a threshold of 0.67 mmol/L with a specificity of 80.0% (95% CI: 51.90–95.70%) and sensitivity of 65.98% (95% CI: 55.70–75.30%), the negative predictive value and negative likelihood ratio were 26.70 (95% CI: 14.60–41.90) and 0.43 (95% CI: 0.30–0.60), correspondingly.

## Discussion

Cx43 protein performs a vital function in the genesis, invasion, and metastasis of tumors. Cx43 is synthesized in ribosomes attached to the endoplasmic reticulum and is transported via the Golgi to the cell membrane to perform its function as a linker [32]. Phosphorylation of Cx43 confers specific functions. The abnormal expression or localization of Cx43 is related to the alteration of homogeneous or heterogeneous gap junction intercellular communication in malignant cells. Studies have confirmed that the mechanism of the decrease of intercellular junction protein in tumor cells may be related to gene silencing, gene mutation, and post-translational modification [33]. Tang et al. found that the expression of Cx43 was reduced in 78.3% of patients with primary gastric cancer, and the decreased expression of Cx43 was linked to advanced lymph node metastasis and clinical stage. Low expression of Cx43 was proved to be beneficial to the progression of primary gastric cancer [34]. Dominguez et al. confirmed that the aberrations of Cx43 expression are associated with thyroid papillary carcinoma [35]. Caltabiano et al. also suggested that the loss of functional Cx43 would lead to more malignant phenotypes in astrocytic brain tumors [36].

Wang et al. showed that Cx43 expression was lower in melanoma than in human epidermal melanocytes [37]. The overexpression of Cx43 significantly inhibits the proliferation as well as colony formation of melanoma cells in vitro. However, the effect of Cx43 knockout on cell proliferation and colony formation is reversed. Bioinformatics prediction and luciferase-reported gene assay have shown that miR-106a targets the 3′ untranslated regions of Cx43 and regulates its mRNA and protein expression levels in melanoma cells. The expression level of miR-106a is upregulated in melanoma cells, and its overexpression attenuates the effect of upregulated expression of Cx43. Ableser et al. found that Cx43 plays an anticancer role in the development of melanoma [38]. Numerous research reports have confirmed that exosomes are vesicle-like bodies secreted by cells that can help the immune escape of tumor cells and promote tumor cell metastasis [39–41]. Cx43 can be used as a marker for differential diagnosis of tumors. Exosomes can activate the immune system to inhibit tumor development, and can also be used as a potential natural carrier to deliver miRNA and chemotherapeutic drugs to tumor cells. To date, the expression, as well as prognostic value of exosomes in melanoma, are yet to be elucidated. The current research is thought to be the first to investigate about the prospective use of plasma exosome-derived CX43 in the diagnosis of melanoma.

In this study, we isolated plasma exosome-derived Cx43 from patients with melanoma and healthy control subjects and identified them with TEM, NTA, and WB. TEM illustrated that the background was clear, exosomes aggregated and distributed in the field of vision, with relatively uniform and relatively full size. The diameter ranged from 100 to 200 nm. The shape of exosome was a double disc-like vesicle structure with a full lipid envelope. The overall particle median size was around 100 nm, with the majority of the particles falling between 50 and 200 nm. NTA showed that only a few particles were distributed within the range of 0 to 50 nm in diameter, and no diameter exceeded 300 nm. Western blot suggested that CD63, CD9, and TSG101 were positive in the exosomal cohort. The results indicated that exosomes can be successfully isolated and used in the subsequent experiments of this study.

The levels of plasma exosome-derived Cx43 were subsequently contrasted among patients with melanoma and healthy control subjects, which demonstrated a substantial downregulation in plasma exosome Cx43 in melanoma patients. KM analysis illustrated that melanoma patients who exhibited lower levels of plasma exosome-derived Cx43 had poorer OS and DFS. We also examined the associations between levels of plasma exosome-derived Cx43 and pathological features in individuals with melanoma. There was no significant difference in sex or age in the plasma exosome-derived Cx43 levels between the two cohorts. Nevertheless, the levels of plasma exosome-derived Cx43 in patients with melanoma whose tumor was situated in the skin, with a size of < 10 cm, Clark level I–III, TNM stage IIb–IV, and had no lymph node metastasis were considerably elevated as opposed to patients with a tumor located in the viscera or mucosa, with a size of ≥ 10 cm, Clark level IV–V, TNM stage IIb–IV and had lymph node metastasis.

In melanoma patients, we examined more precisely the prognostic significance of the plasma exosome-derived Cx43. The ROCs of plasma-exosome-derived Cx43 were 0.78 (95% CI: 0.70–0.86) and 0.77 (95% CI: 0.66–0.84), correspondingly, to forecast 5-year OS and DFS for patients with melanoma. All the preceding data demonstrate that it has success in forecasting five-year DFS and five-year OS of melanoma patients.

## Conclusion

Our study demonstrated that the levels of plasma exosome-derived Cx43 in patients with melanoma were considerably downregulated. The patients with melanoma exhibiting lower levels of plasma exosome-derived Cx43 were found to have poorer OS and DFS. This implies that plasma exosome-derived Cx43 levels could function as a prospective prognostic indicator for 5-year OS and 5-year DFS of patients with melanoma.

## Electronic supplementary material

Below is the link to the electronic supplementary material.

Supplementary Material 1

## Data Availability

All data relevant to the study are included in the article.

## Abbreviations

Cx43: Connexin43
NTA: Nanoparticle tracking analysis
TEM: Transmission electron microscopy
VEGF: Vascular endothelial growth factor
DFS: Disease-free survival
OS: Overall survival.
