# Supplementary figures and images for: Plasma exosome-derived connexin43 as a promising biomarker for melanoma patients
Source: BMC Cancer. 2023 Mar 14;23:242. doi: 10.1186/s12885-023-10705-9 (PMC10012581; doi:10.1186/s12885-023-10705-9)

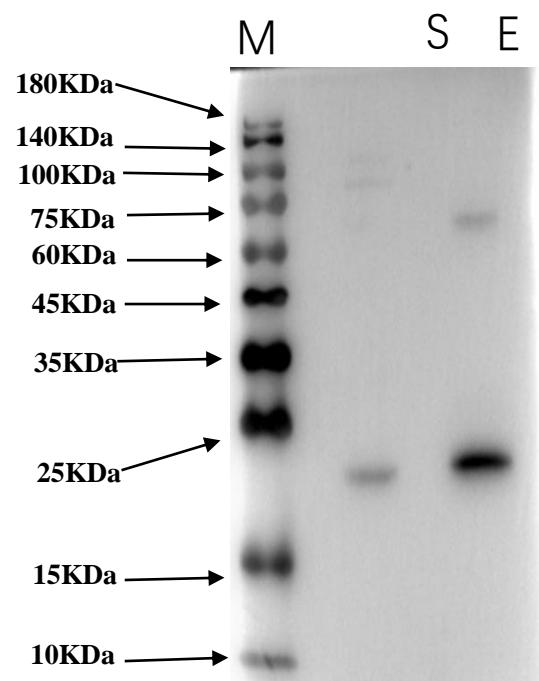

CD9,25KDa,30S

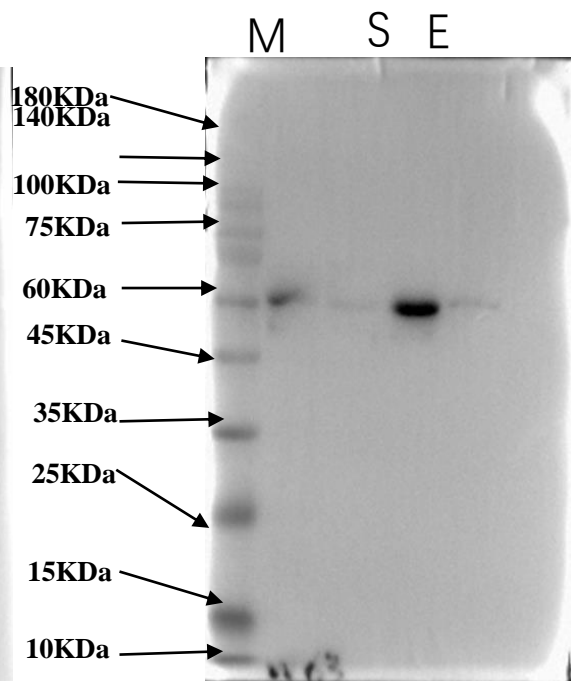

CD63,53KDa,150S

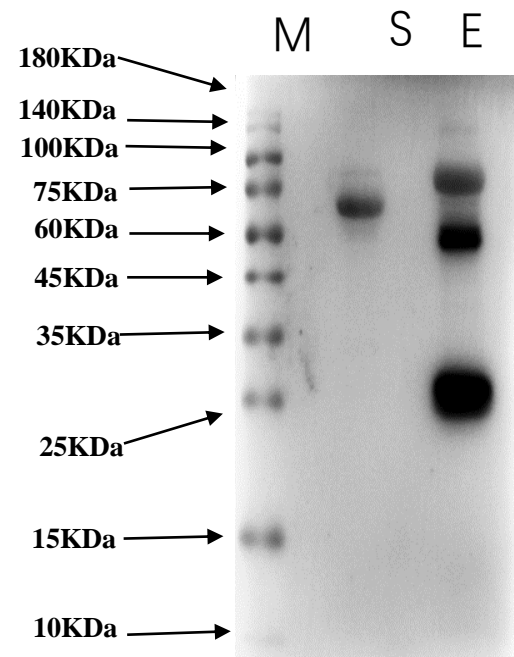

TSG101,44KDa,30S

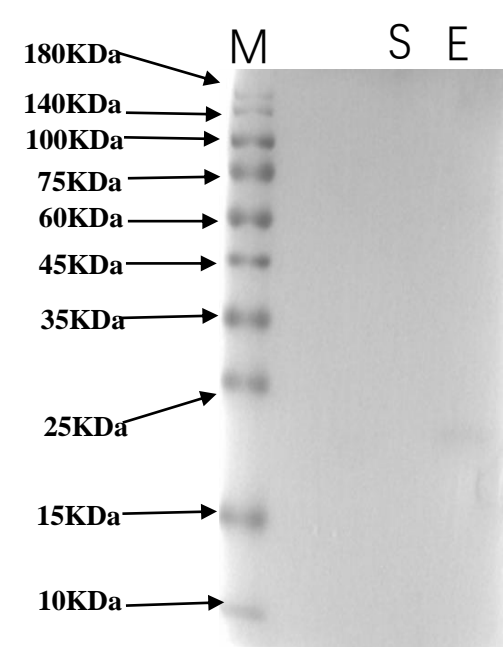

Calnexin,94KDa,60S

Supplement: Supplementary file 1 — Supplementary Material 1 [file 12885_2023_10705_MOESM1_ESM.pdf]
